# Unintended impact of COVID-19 pandemic on the rate of catheter related nosocomial infections and incidence of multiple drug resistance pathogens in three intensive care units not allocated to COVID-19 patients in a large teaching hospital

**DOI:** 10.1186/s12879-022-07962-7

**Published:** 2023-01-06

**Authors:** Farid Zand, Hedayatollah Vakili, Naeimehossadat Asmarian, Mansoor Masjedi, Golnar Sabetian, Reza Nikandish, Elham Shafiee, Azita Tabatabaei Esfehani, Fatemeh Azadi, Anahita Sanaei Dashti

**Affiliations:** 1 Anesthesiology and Critical Care Research Center, Shiraz University of Medical Sciences, Shiraz, Iran; 2 Faghihi Hospital, Shiraz University of Medical Sciences, Shiraz, Iran; 3 Trauma Research Center, Shiraz University of Medical Sciences, Shiraz, Iran; 4 Nemazee Hospital, Shiraz University of Medical Sciences, Shiraz, Iran; 5 Professor Alborzi Clinical Microbiology Research Center, Shiraz University of Medical Sciences, Shiraz, Iran

**Keywords:** Nosocomial infection (NI), Intensive care unit (ICU), Ventilator associated pneumonia (VAP), Central line associated blood stream infection (CLABSI), Catheter-associated urinary tract infections (CAUTI), Multiple drug resistance (MDR), COVID-19

## Abstract

**Background:**

The prevalence of resistant hospital infections in the intensive care unit (ICU) increases mortality and antibiotic resistance. COVID-19 pandemic may have unintended impact on nosocomial infections (NI) and the prevalence of resistant microorganism.

**Methodology:**

The present non-interventional study was performed by a pre and a post survey each lasting 8 months before (March–October 2019) and after (March–October 2020) the onset of COVID-19 pandemic in three ICU’s, not allocated to COVID-19 patients, in Nemazee Hospital, Shiraz, Iran. The rates of the following nosocomial infections were compared at pre- and post-pandemic period: ventilator associated pneumonia (VAP), central line associated blood stream infection (CLABSI), catheter-associated urinary tract infections (CAUTI) and incidence of multiple drug resistance (MDR) pathogens.

**Results:**

Pre-pandemic and pandemic incidence of VAP was 23.5 and 17.2 cases per 1000 device-days, respectively; an absolute decrease of 27%. The main reason for the decrease in the rate of VAP during the pandemic was a significant decrease in the rate of VAP caused by *Acinetobacter baumannii*; from 39 to 17% in total VAP episodes. The rate of VAP associated with other microorganisms remained relatively unchanged from 14.2 cases in pre-pandemic period to 14.3 cases per 1000 MV-days during the pandemic (P = 0.801). Pre-pandemic incidence of CLABSI was 7.3 cases and, in pandemic period, was 6.5 cases per 1000 device-days (IRR = 0.88, 95% CI  0.43–1.73, P = 0.703). Pre-pandemic incidence of CAUTI was 2 and in pandemic period, was 1.4 cases per 1000 device-days (IRR = 0.70, 95% CI  0.22–1.98, P = 0.469).

**Conclusion:**

The results of the present study showed a decrease in the incidence of VAP in critically ill non-COVID-19 patients during the pandemic compared to before the pandemic, especially regarding *Acinetobacter baumannii*.

**Supplementary Information:**

The online version contains supplementary material available at 10.1186/s12879-022-07962-7.

## Introduction

Nosocomial Infection (NI) as an infectious event that is diagnosed > 48 h after admission without evidence that the pathogen was already in the incubation phase [1]. NIs account for 4% of all hospital admissions, of which 9–20% are in the intensive care unit (ICU) [2, 3]. They have devastating consequences such as increased morbidity, mortality, lifelong disability and financial burden for the patients and health system. Thus, infection prevention and control (IPC) would be an essential task to decrease NIs at the hospitals and particularly in the ICUs [4].

While the number of patients admitted to ICUs is less than that of other wards, it seems the incidence of nosocomial infections is higher due to the long duration of admission. Various factors could affect the rate and type of nosocomial infections in the ICU, including the existing etiologic pathogens, novel or emerging pathogens, host risk factors, antimicrobial resistance pattern and current prevention strategies. Increasing virulence and antimicrobial resistance of nosocomial infections mandate increasing efforts toward their prevention [5].

Since the end of 2019, the world has faced COVID-19 pandemic that has heavily affected the healthcare system worldwide. COVID-19 is primarily transmitted through respiratory droplets and close contact, and all individuals are susceptible to the disease [6]. Due to the high transmission rate of COVID-19 and the urgent need to protect patients admitted to the wards, efficient IPC strategies are necessary [7]. Although prior to the COVID-19 pandemic IPC was considered in hospitals, but these strategies were mostly deployed in high-risk units, given concerns with sustainability and cost-effectiveness. For NI to be prevented in the hospitals, infection control standards recommend implementing strong triage and screening strategies, standard precautions such as hand hygiene and personal protective equipment usage, strengthening environmental health and crowding reduction [8, 9].

A number of studies have investigated the changes in prevalence of NI at the pre and post COVID-19 outbreak era [10, 11]. Factors such as adherence to infection control protocols, proper training and observance of infection control protocols, raising awareness, educating staff, hand hygiene, contact precautions, disinfection of the environment, and finally elimination of infection using antimicrobial agents affect the preventing nosocomial infections [9].

In Iran, along with the rest of the world, extensive measures were taken to prevent the spread of this disease in the society and in medical settings [12]. Nemazee hospital was one of the leading medical centers that strengthened infection control at all wards. The present study was designed to investigate unintended impact of COVID-19 pandemic on the rate of nosocomial infections including ventilator associated pneumonia (VAP), central line associated blood stream infection (CLABSI), catheter-associated urinary tract infections (CAUTI) and prevalence of multiple drug resistance (MDR) pathogens in three ICU’s not allocated to COVID-19 patients in Nemazee hospital in 2020. The aim of this study was to evaluate the effect of controlling infection programs implemented due to pandemic outbreaks in the ICU wards to prevent resistant infections.

## Patients and methods

### Study design and setting

The study protocol enrolled all the patients over 18 years old admitted to General, Central and Emergency ICU’s of Shiraz Nemazee hospital. These wards were not allocated to COVID-19 in 2020. Nemazee hospital is a university hospital with more than 900 beds, affiliated to Shiraz University of Medical Sciences. It is a tertiary care center and a large referral center in south of Iran. General, Central and Emergency ICU have 31 beds and admit adult patients. The study protocol was approved by the ethical committee of Shiraz University Medical Science (IR.sums.med.rec.1400.187). The present study was a pre and post-survey in two time periods of eight months before and after the onset of COVID-19 pandemic; March to October 2019 and March to October 2020 in three ICU’s.

The inclusion criterion was non- COVID patients who were admitted to the Central, Emergency and General ICU wards during the study period in Nemazee hospital. The exclusion criteria were patients under 18 years old, brain death and who died in less than 72 h in ICU. The incidence of the following nosocomial infections was compared before and during the pandemic; ventilator associated pneumonia (VAP), central line associated blood stream infection (CLABSI), catheter-associated urinary tract infections (CAUTI). Also the prevalence of multiple drug resistance (MDR) pathogens in these ICU’s were compared before and during the pandemic for an 8-month period. Both infection during the ICU stay and colonization on arrival to ICU by MDR pathogens were recorded. Surveillance cultures were taken for every patient with history of recent hospitalization for more than 2 days, when the patients entered the ICU.

Diagnosis of VAP, CLABSI, and CAUTI was based on positive cultures for each and other diagnostic symptoms based on the latest guidelines from the Centers for Disease Control and Prevention (CDC). The data regarding incidence of these infections were prospectively collected by a trained nurse not involved in the care of the patients. Our hospital has joined the International Nosocomial Infection Control Consortium (INICC) since 2014. This is an international collaboration of more than 200 health centers in more than 50 countries for standard reporting and prevention of hospital acquired infections. The collected data are uploaded in a central software on a daily basis where the quality of data is checked routinely by an expert team. The necessary data that was not present in INICC database were collected from either electronic medical records of the patients or the Iran Intensive Care Registry (IICUR). This is a system of prospective data gathering in Nemazee hospital which is in place since 2017, in collaboration with Australia and New Zealand Intensive Care Society (ANZICS).

The effectiveness of infection prevention and control programs such as the use of personal protective equipment such as masks, gauze, gloves and frequent hand washing, which has increased due to the observance of the treatment staff during the outbreak of the COVID-19, were also examined. Adherence to hand hygiene was checked and recorded by the nurses in charge of infection control at the hospital. In the fields of hand hygiene, the basics of infection control, environmental health and the necessary training equipment were given to the staff by the nurses in charge of hospital education. During the outbreak of COVID-19, surfaces were cleaned daily using a hypochlorite-based disinfectant solution of 1:1000. Also, according to the IPC program of the Infection Control Unit of the Nemazee hospital, all health care providers in ICU, had to use N95 masks, gowns, gloves and face shields when contacting patients as well as observing the principles of frequent hand washing as standard.

### Statistical analysis

In this study, continuous variables were presented as mean ± standard deviation, median (interquartile range) and categorical variables as number and percentage. The continuous and categorical variables were compared between two periods by Mann–Whitney U test and chi-square. Comparisons of incidence rates were made using the incidence rate ratio (IRR) method for Poisson distribution. We calculated the IRR of incident event by dividing the number of incident by the number of “device-days” or “patient-days” in each period for counting variables. We considered the statistical significance of the difference between the IRR CI for two given periods, by calculating the Z-value [Z = (ln(IR1)—ln(IR2))/sqrt(SD1*SD1 + SD2*SD2)]. Z ≤ − 1.96 or Z ≥ 1.96 were considered statistically significant. Data analysis was performed by Stata 16 (StataCorp, College Station, Texas) and a P-value (2-tailed) of ≤ 0.05 was considered significant.

### Nosocomial infection (NI)

Nosocomial Infection (NI) as an infectious event that is diagnosed > 48 h after admission without evidence that the pathogen was already in the incubation phase and whose diagnosis is based on CDC criteria [1].

### Ventilator associated pneumonia (VAP)

VAP is hospital-acquired lung infection in a patient who has been under mechanical ventilation for more than 48 h and whose diagnosis is based on CDC criteria [13].

### Central line associated blood stream infection (CLABSI)

Positive blood culture associated with clinical presentation of infection in a patient with a central venous catheter who has no other source of bacteremia and more than 48 h have passed since the catheter was implanted [14].

### Catheter-associated urinary tract infections (CAUTI)

Patients of any age who have had a urinary catheter for two days or more and with one of the following symptoms:

- Fever: more than 38 °C without a specific cause.
- Localized pain in the suprapubic area for no apparent reason.
- Localized tenderness in the suprapubic region for no apparent reason.
- Frequent urination for no apparent reason within 48 h of catheter removal.
- Urinary incontinence for no apparent reason.
- Urgency of urination without a specific cause with.
- Positive urine culture more than 10^5^ cfu/ml which do not grow more than two species of microorganisms [14].

### Multiple drug resistance (MDR)

MDR was defined as acquired non-susceptibility to at least one agent in three or more antimicrobial categories [15].

### Extensively drug-resistant (XDR)

XDR was defined as non-susceptibility to at least one agent in all but two or fewer antimicrobial categories (i.e., bacterial isolates remain susceptible to only one or two categories) [15].

### Hand hygiene

Adherence to hand hygiene according to the hand hygiene bundle of Nemazee Hospital (WHO module based), including five moments of hand hygiene.

## Results

The number of admitted cases in pre COVID-19 period were 566 patients and during the pandemic period were 525 patients. Mean of age (53.72 ± 19.17 and 52.31 ± 19.53) and proportion of gender [306(54.1) for females and 272(51.8) for males] were the similar in pre pandemic and pandemic period**.** Percentage of medical (non-surgical) admission were lower in pandemic period than pre pandemic (51% vs. 62%). The major clinical information of the patients presented in Table 1.

**Table 1:** Clinical characteristics of ICU patients

	Before Covid-19 (2019) N = 566	During Covid-19 (2020) N = 525	P-value
ICU LOS1, days, median (IQR)	5(3–14)	4(2–8)	< 0.001
Number of bed day in ICU	6029	3547	< 0.05
MV2 days	6(2–14)	3(2–8)	< 0.001
Central venous catheter days	7(4–16)	5(3–11)	0.001
urinary catheter days	6(3–15)	4.5(3–9)	< 0.001
Apache score П	20(13–26)	17(10–23)	< 0.001
Mortality rate	68(12)	50(9.5)	0.317
VAP, n (%)	89(16)	36(7)	< 0.001
Utility ratio (MV days/patient days) * 100	62.81	59.09	0.208
CLABSI, n (%)	26(4.6)	15(2.9)	0.132
Utility ratio (CL days/patient days) * 100	58.99	65.46	0.028
CAUTI, n (%)	13(2.3)	6(1.1)	0.145
Utility ratio (UC days/patient days) * 100	100	100	> 0.999

Length of stay (LOS), Mechanical Ventilation (MV2), Apache: Acute physiology and chronic health evaluation II Ventilator Associated Pneumonia (VAP), Central line Associated Blood Stream Infection (CLABSI), Catheter-associated Urinary Tract Infections (CAUTI)

Utility ratio was defined as the number of days when an especial catheter or mechanical ventilation was used in the patient, divided by the total days of the patients stay in the ICU.

### VAP

**Fig. 1:**
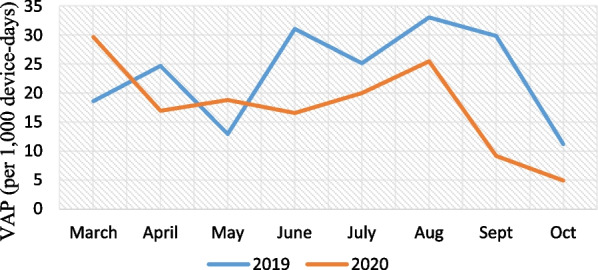
VAP (per 1000 device-days)

Pre-pandemic, the incidence of VAP was 23.50 cases per 1000 device-days (89 cases; 3787 device-days) and, in pandemic period, was 17.18 cases per 1000 device-days (36 cases; 2096 device-days), that the difference between them weren’t statistically significant (IRR = 0.73, 95% CI  0.48–1.09, P = 0.111) (Fig. 1).

**Fig. 2:**
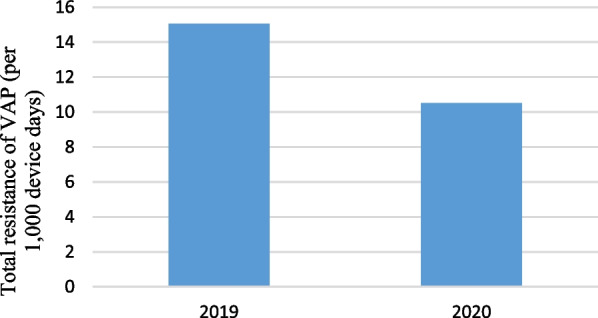
Ventilator associated pneumonia caused by MDR microorganisms (per 1000 device-days)

Pre-pandemic, the rate of VAP caused by MDR microorganisms (both gram positive and non *Acinetobacter baumannii* gram negatives) was 15.05 cases per 1000 device-days (57 cases; 3787 device-days) and, in pandemic period, was 10.5 cases per 1000 device-days (22 cases; 2096 device-days) (IRR = 1.43, 95% CI  0.86–2.46, P = 0.148) (Fig. 2).

**Fig. 3:**
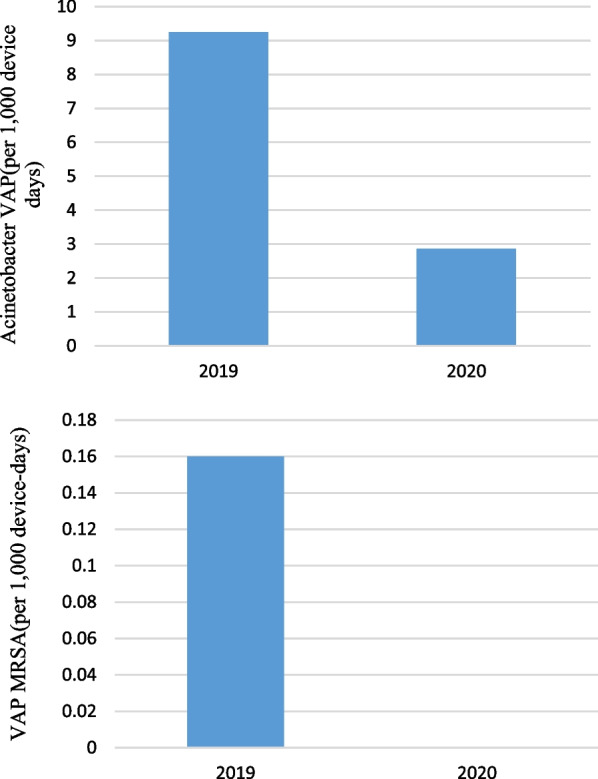
Acinetobacter and MRSA VAP (per 1000 device-days)

Pre-pandemic, the rate of *Acinetobacter baumannii* gram negatives VAP was 9.24 cases per 1000 device-days (35 cases; 3787 device-days) and, in pandemic period, was 2.86 cases per 1000 device-days (6 cases; 2096 device-days) that the difference was statistically significant (IRR = 3.23, 95% CI  1.34–9.39, P = 0.005). Pre-pandemic, the rate of MRSA VAP was 0.26 cases per 1000 device-days (1 case; 3787 device-days) and, in pandemic period, was 0 cases per 1000 device-days (0 cases; 2096 device-days) (P = 0.456) (Fig. 3).

### CLABSI

**Fig. 4:**
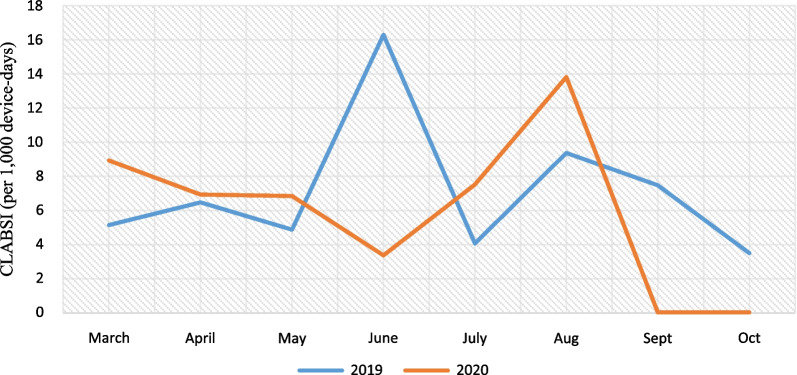
CLABSI (per 1000 device-days)

Pre-pandemic, the incidence of CLABSI was 7.30 cases per 1000 device-days (26 cases; 3557 device-days) and, in pandemic period, was 6.45 cases per 1000 device-days (15 cases; 2322 device-days) that the difference between them weren’t statistically significant (IRR = 0.88, 95% CI  0.43–1.73, P = 0.703) (Fig. 4).

**Fig. 5:**
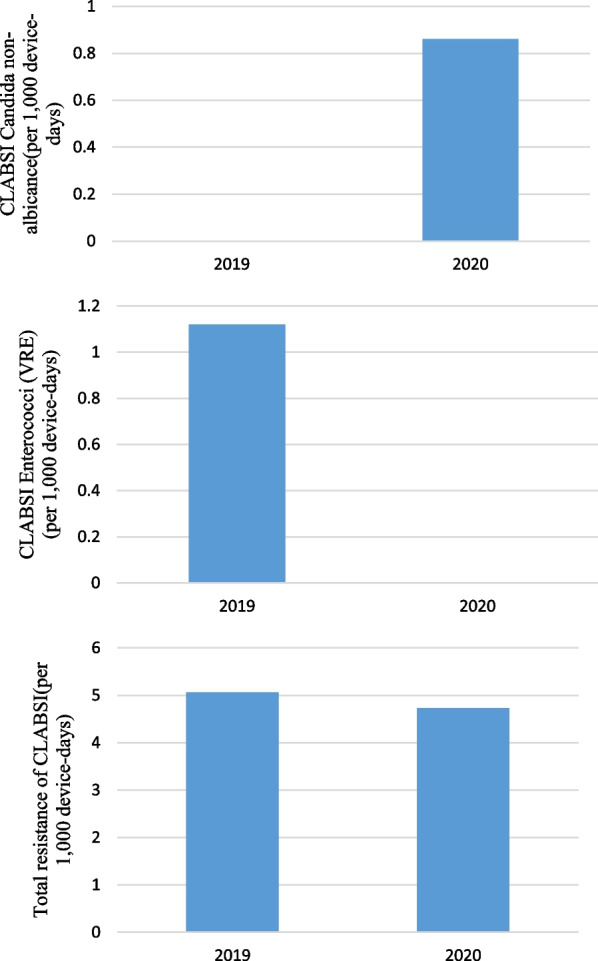
CLABSI caused by Enterococci (VRE), Candida non-albicans and Total resistance of CLABSI (gram positive, gram negatives Non *Acinetobacter baumannii* and *Acinetobacter baumannii* gram negatives) (per 1000 device-days)

Pre-pandemic, the rate of Candida non-albicans for CLABSI was 0 cases per 1000 device-days (0 cases; 3557 device-days) and, in pandemic period, was 0.86 cases per 1000 device-days (2 cases; 2322 device-days) that the difference between them weren’t statistically significant (P = 0.080). Pre-pandemic, the rate of Enterococci (VRE) for CLABSI was 1.12 cases per 1000 device-days (4 cases; 3557 device-days) and, in pandemic period, was 0 cases per 1000 device-days (0 cases; 2322 device-days) that the difference between them weren’t statistically significant (P = 0.106). Pre-pandemic, the rate of total resistance of CLABSI (gram positive, *non Acinetobacter baumannii* gram negatives and *Acinetobacter baumannii* gram negatives) was 5.06 cases per 1000 device-days (18 cases; 3557 device-days) and, in pandemic period, was 4.73 cases per 1000 device-days (11 cases; 2322 device-days) (IRR = 1.06, 95% CI  0.47–2.50, P = 0.863) (Fig. 5).

**Fig. 6:**
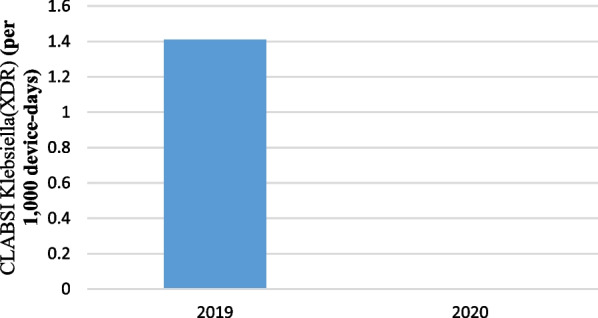
CLABSI caused by Klebsiella (XDR) (per 1000 device-days)

Pre-pandemic, the rate of Klebsiella gram negatives (XDR) for CLABSI was 1.41 cases per 1000 device-days (5 cases; 3557 device-days) and, in pandemic period, was 0 cases per 1000 device-days (0 cases; 2322 device-days) that the difference between them weren’t statistically significant (P = 0.070) (Additional file 1) (Fig. 6).

**Fig. 7:**
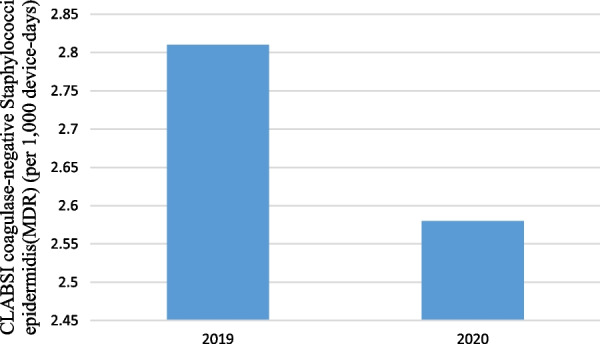
CLABSI caused by coagulase-negative Staphylococci epidermidis (MDR) (per 1000 device-days)

Pre-pandemic, the rate of coagulase-negative Staphylococci epidermidis(MDR) for CLABSI was 2.81 cases per 1000 device-days (10 cases; 3557 device-days) and, in pandemic period, was 2.58 cases per 1000 device-days (6 cases; 2322 device-days) that the difference between them weren’t statistically significant (IRR = 1.09, 95% CI  0.36–3.64, P = 0.870) (Fig. 7).

**Fig. 8:**
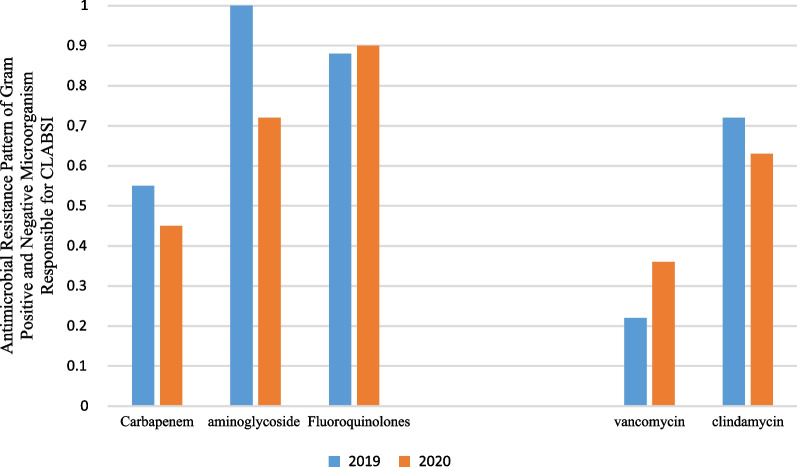
Antimicrobial Resistance Pattern of Gram Positive and Negative Microorganism Responsible for CLABSI

Pre-pandemic, the rate of Antimicrobial Resistance Pattern of Gram Positive and Negative Microorganism Responsible for CLABSI: (1) Gram Negative antimicrobial (Carbapenem, aminoglycoside and Fluoroquinolone), and (2) Gram Positive antimicrobial (Vancomycin, Clindamycin) in comparison of pandemic period which was reported difference between them and weren’t statistically significant except for Aminoglycoside P = 0.021 (Fig. 8).

### CAUTI

**Fig. 9:**
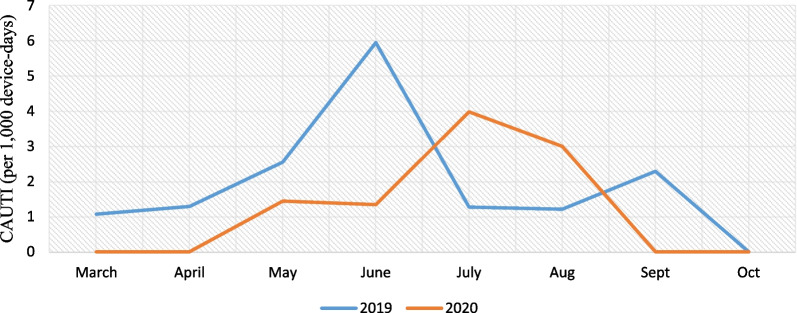
CAUTI (per 1000 device-days)

Pre-pandemic, the incidence of CAUTI was 1.99 cases per 1000 device-days (13 cases; 6535 device-days) and, in pandemic period, was 1.39 cases per 1000 device-days (6 cases; 4303 device-days) that the difference between them weren’t statistically significant (IRR = 0.70, 95% CI 0.22–1.98, P = 0.469) (Fig. 9).

**Fig. 10:**
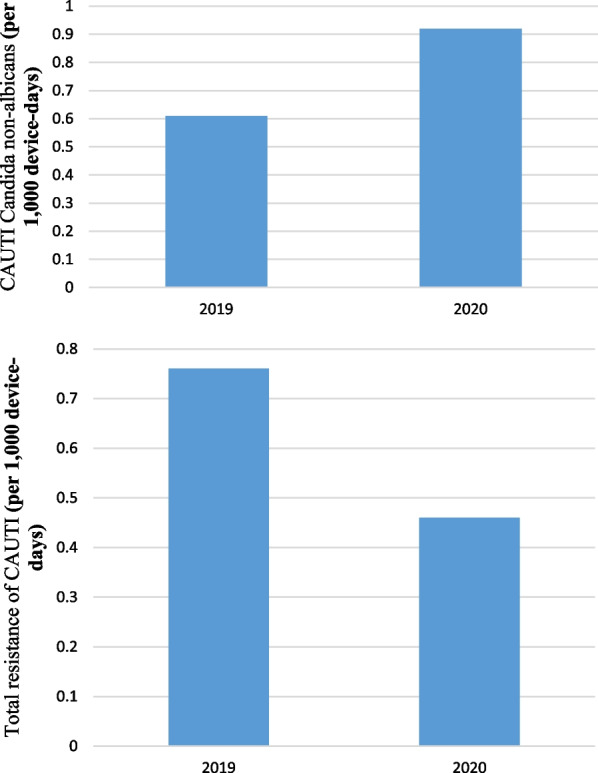
Total resistance of CAUTI (gram positive, gram negatives *non Acinetobacter baumannii*, *Acinetobacter baumannii* gram negatives) and Candida non-albicans (per 1000 device-days)

Pre-pandemic, the rate of Candida non-albicans for CAUTI was 0.61 cases per 1000 device-days (4 cases; 6535 device-days) and, in pandemic period, was 0.92 cases per 1000 device-days (4 cases; 4303 device-days) that the difference between them weren’t statistically significant (IRR = 0.66, 95% CI  0.12–3.53, P = 0.552). Pre-pandemic, the rate of total resistance of CAUTI (gram positive, gram negatives *non Acinetobacter baumannii*, *Acinetobacter baumannii* gram negatives) was 0.76 cases per 1000 device-days (5 cases; 6535 device days) and, in pandemic period, was 0.46 cases per 1000 device-days (2 cases; 4303 device-days) that the difference between them weren’t statistically significant (IRR = 1.64, 95% CI  0.27–7.13, P = 0.547) (Fig. 10).

### Arrival time and during ICU stay colonization

Pre-pandemic, the rate of Enterococci (VRE) for Arrival time colonization was 1.49 cases per 1000 patient-days (9 cases; 6029 patient-days) and, in pandemic period, was 0.85 cases per 1000 patient-days (3 cases; 3547 patient-days) that the difference between them weren’t statistically significant (IRR = 1.76, 95% CI  0.48–6.51, P = 0.388). Pre-pandemic, the rate of total resistance of Arrival time colonization (gram positive, gram negatives *non Acinetobacter baumannii*, *Acinetobacter baumannii* gram negatives) was 5.97 cases per 1000 patient-days (36 cases; 6029 patient-days) and, in pandemic period, was 7.89 cases per 1000 patient-days (28 cases; 3547 patient-days) that the difference between them weren’t statistically significant (IRR = 0.77, 95% CI  0.45–1.29, P = 0.266). Pre-pandemic, the rate of MRSA for Arrival time colonization was 0.16 cases per 1000 patient-days (1 cases; 6029 patient-days) and, in pandemic period, was 0 cases per 1000 patient-days (0 cases; 3547 patient-days) (P = 0.443).

**Fig. 11:**
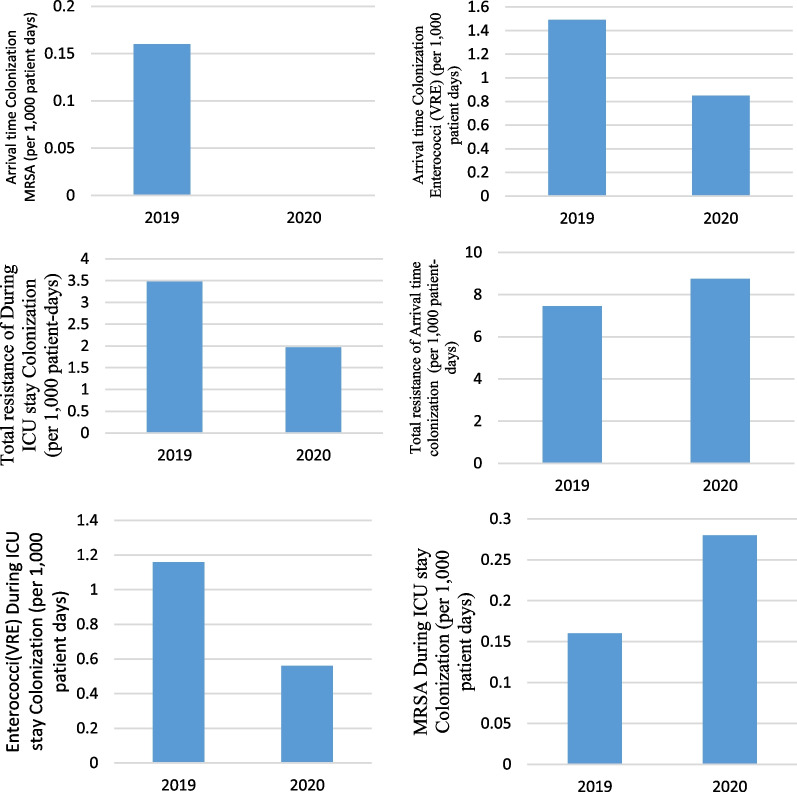
Colonization present at the time of admission to the ICU caused by Enterococci (VRE), Total resistance of arrival time, Arrival time MRSA Colonization, Enterococci(VRE) During ICU stay Colonization, Total resistance of during ICU stay Colonization, MRSA during ICU stay Colonization (per 1000 patient days)

Pre-pandemic, the rate of Enterococci for During ICU stay Colonization was 1.16 cases per 1000 patient-days (7 cases; 6029 patient-days) and, in pandemic period, was 0.56 cases per 1000 patient-days (2 cases; 3547 patient-days) that the difference between them weren’t statistically significant (IRR = 2.05, 95% CI  0.43–9.91, P = 0.357). Pre-pandemic, the rate of total resistance of During ICU stay Colonization (gram positive, gram negatives non *Acinetobacter baumannii*, *Acinetobacter baumannii* gram negatives) was 3.48 cases per 1000 patient-days (21 cases; 6029 patient-days) and, in pandemic period, was 1.97 cases per 1000 patient-days (7 cases; 3547 patient-days (IRR = 1.76, 95% CI  0.72–4.91, P = 0.187). Pre-pandemic, the rate of MRSA for During ICU stay Colonization was 0.16 cases per 1000 patient-days (1 cases; 6029 patient-days) and, in pandemic period, was 0.28 cases per 1000 patient-days (1 cases; 3547 patient-days) that the difference between them weren’t statistically significant (IRR = 0.58, 95% CI  0.04–9.40, P = 0.704) (Fig. 11).

### Personal protective equipment (PPE)

PPE compliance was utilized by extracting the hospital information system (HIS). Routine use of surgical mask and N95 mask was increased from zero in pre-pandemic period to 77% during the pandemic. Also, surgical gown compliance was 70.75% before COVID-19 group and 77% during COVID-19 group. Regarding hand hygiene as an important measure of infection prevention hand hygiene compliance was 50.82% before COVID-19 group and 66.15% during COVID-19 group. Also, Alcoholic hand rub consumption (liters per month) was 18.37 before COVID-19 group and 14.12 during COVID-19 group (Table 2).

**Table 2:** Personal protective equipment (PPE) before Covid-19 and during Covid-19

PPE	Before Covid-19 (2019) N = 566	During Covid-19 (2020) N = 525	P-Value
Surgical and N95 mask compliance (% per month)	0	77	< 0.001
Surgical gown compliance (% per month)	70.75	77	NS
Hand hygiene			
Hand hygiene compliance (% of months)	50.82	66.15	NS
Hand rub alcohol consumption (liters per month)	18.37	14.12	NS
Environmental health			
Hydrogen peroxide consumption (liters per month)	13.5	17.12	NS
Liquid soap consumption (liters per month)	13	17	NS
Training (hours/personnel)			
Hand hygiene	3	87	< 0.001
Basics of Infection Control	300	354	0.034
Environmental health (surfaces, equipment)	0	72	< 0.001

## Discussion

The data from ICUs, comparing a period before COVID-19 pandemic and during the pandemic showed that VAP incidence was decreased in during COVID-19 period, especially the VAP caused by *Acinetobacter baumannii* species. The main strategies to combat *Acinetobacter baumannii* outbreaks includes health personnel education regarding infection control topics, increasing hand hygiene compliance, strict isolation precautions and appropriate environmental cleaning [16]. Thus,the significant reduction in the VAP caused by *Acinetobacter baumannii* in our center has explanations like increased staff education toward infection control basics, increased PPE usage, more hand hygiene compliance, decrease in median ICU stay and reduced mean ventilation days during the COVID-19 pandemic.

Extensive training of health care personnel was dramatically increased during COVID-19 as the main policy to prevent COVID-19 extension. Basics of infection control, including isolation precautions, PPE usage and specially hand hygiene technics were instructed to the staff of all categories, according to WHO modules.

Hand hygiene is the most important strategy to reduce nosocomial infections. In our survey hand hygiene compliance increased from 50% before pandemic to 66% during pandemic. The rate of VAP associated with other microorganisms remained relatively unchanged from 14.2 cases in pre-pandemic period to 14.3 cases per 1000 MV-days during the pandemic (P = 0.801). Our explanation is the relatively small number of the VAP episodes caused by microorganisms other than *Acinetobacter baumannii*.

McMullen and his colleagues in ‘infection control and epidemiology’ stated some predictions about “the impact of SARS-CoV-2 on hospital acquired infection rates in the United States” [17]. The followings are the main content of their statements: due to application of a series of waivers and exceptions in infection control rules, to make more flexibility for healthcare workers involving in COVID-19 crisis, it is anticipated that CLABSI and CAUTI increases. The utmost impact would occur on CLABSI rates. Accordingly two of their facilities have had increases rates of CLABSI during COVID-19. Hospital A faced a 420% increase to rate = 5.38 cases per 1000 central line days, while Hospital B faced a 327% increase to rate = 3.79 cases per1, 000 central line days). Based on the authors’ viewpoint, several factors are expected to increase the number of CLABSI cases, though decreasing low-risk central line denominators, resulting in overall increases in rates.

A comparative retrospective cohort study in a tertiary care hospital in Detroit within a “pre- COVID-19” and “COVID-19” time interval, was done. Data obtained from Infection Control Surveillance System showed that the CLABSI rate per 1000 line days increased from 0.71 in pre-COVID-19 time frame to 2.70 during COVID-19 (P-value < 0.01). No explanation was made by the authors for this 280% increase in CLABSI rate [18].

In a large study in USA, researchers analyzed the CLABSIs, CAUTIs, ventilator associated events (VAEs), surgical site infections, and Clostridioides difficile and methicillin-resistant Staphylococcus aureus (MRSA) bacteremia episodes that were reported to the National Healthcare Safety Network during 2019 and 2020 by hospitals [19].

A significant increase in the national rate of CLABSI, CAUTI, VAE, and MRSA bacteremia were detected in 2020 with the largest rise for CLABSI occurrence. The investigators attributed the increased rate of device-associated infections during the pandemic to factors like increased length-of-stay of patients, admission of more severely ill patients with further comorbidities and longer duration of device use. Furthermore they reported an increased rate of ventilator-associated conditions in critically ill COVID-19 patients [20, 21]. They concluded that it is necessary to maintain conventional infection prevention and control practices.

In our study, the rate of CLABSI and CAUTI, were also decreased, though not statistically significant; this could be due to small number of cases. This result was somehow predictable too, due to a decrease in central venous catheter and urinary catheter days, respectively and increased usage of PPE and more hand hygiene practice.

A retrospective study conducted to compare the prevalence of NIs before and after COVID-19 pandemic for 6 months in a children hospital in China showed a decrease in the rate of nosocomial infections: The rate of reduction for VAP was significant and for CAUTI and CLABSI were not statistically significant. Interestingly, the rate of nosocomial infections of the ICU increased from 5 to 6.20%. There was a significant increase in the rate of appropriate hand washing, the number of protective gloves and aprons used per person and the number of healthcare staff per patients [9].

Through a research done in a hospital in southeast Iran, the nosocomial infection rates of critical/intensive care units (CCU/ICUs) and medical-surgical units were assessed during and before the COVID-19 outbreak. There was a 19.75% decrease in the total rate and 39.12% decrease in the CCU/ICUs’ of nosocomial infection during the COVID-19 outbreak (*P* < 0.02). In addition a 19.23% decrease occurred in the total rate of medical-surgical units’ nosocomial infection during the COVID-19 outbreak (*P* < 0.13). Among medical-surgical units, 33.33% and 30.70% decreases were observed in UTI and SSI, respectively [8].

To sum up, the nature and rate of change(s) in nosocomial infections, during an epidemic state, seems to be related not merely to the occurrence of the outbreak, but mostly on factors that lead to nosocomial infections. If hand hygiene compliance, PPE usage, appropriate antiseptic procedures,correct isolation precautions and proper environmental health are increased due to any reason like more ‘infection control basics’ awareness and practice, nosocomial infections rates would decrease and if they are less practiced owning to any reason like crowding and increased work burden, limited infection control items resources, increase in admission of critical patients,less health personnel to patient ratio the nosocomial infections rate would increase.

In our experience, the rate of “education of infection control rules “,and PPE usage were increased,and mean ventilation days,mean admission days, mean central venous catheter days and mean urinary catheter days were decreased, consequently decreasing the rate of VAP, CAUTI and CLABSI.

## Limitation

Lack of personal protective equipment and antiseptics, especially at the beginning of the outbreak of COVID-19, the resistance of some health personnel to use personal protective equipment and non-compliance with health instructions despite the provision of resources and training by the “Infection Control Unit” may affect the results of this study. Also at the time of data collection, patients' personal diseases, such as immune deficiency or cancer, were not recorded, which may affect the difference in catheter related infections between patients of these study groups.

## Conclusion

The amount of VAP caused by *Acinetobacter baumannii* had a significant decrease, but in the cases of VAP caused by resistant organisms, as well as the amount of CLABSI and CAUTI, there was no statistically significant decrease, which was due to the small sample size, and it is possible that interventions such as more education in infection control and increasing adherence to hand hygiene and reduce the duration of connecting unnecessary catheters to be effective.

## Supplementary Information

**Additional file 1.** Additional figures.

## Data Availability

All data generated or analysed during this study are included in this published article.

## Abbreviations

ICU: The intensive care unit
Nis: Nosocomial infections
VAP: Ventilator associated pneumonia
CLABSI: Central line associated blood stream infection
CAUTI: Catheter-associated urinary tract infections
MDR: Multiple drug resistance

## References

[1] Kouchak F, Askarian M (2012). Nosocomial infections: the definition criteria. Iran J Med Sci.

[2] Strich JR, Palmore TN (2017). Preventing transmission of multidrug-resistant pathogens in the intensive care unit. Infect Dis Clin.

[3] Zarb P, Coignard B, Griskeviciene J, Muller A, Vankerckhoven V, Weist K (2012). The European Centre for Disease Prevention and Control (ECDC) pilot point prevalence survey of healthcare-associated infections and antimicrobial use. Eurosurveillance.

[4] Rosenthal VD, Bat-Erdene I, Gupta D, Belkebir S, Rajhans P, Zand F (2020). International Nosocomial Infection Control Consortium (INICC) report, data summary of 45 countries for 2012–2017: device-associated module. Am J Infect Control.

[5] Kollef MH, Torres A, Shorr AF, Martin-Loeches I, Micek ST (2021). Nosocomial infection. Crit Care Med.

[6] Zhou Q, Gao Y, Wang X, Liu R, Du P, Wang X (2020). Nosocomial infections among patients with Covid-19, SARS and MERS: a rapid review and meta-analysis. Ann Transl Med.

[7] Sturdy A, Basarab M, Cotter M, Hager K, Shakespeare D, Shah N (2020). Severe Covid-19 and healthcare-associated infections on the ICU: time to remember the basics?. J Hosp Infect.

[8] Lu D, Wang H, Yu R, Yang H, Zhao Y (2020). Integrated infection control strategy to minimize nosocomial infection of coronavirus disease 2019 among ENT healthcare workers. J Hosp Infect.

[9] Jabarpour M, Dehghan M, Afsharipour G, Hajipour Abaee E, Mangolian Shahrbabaki P, Ahmadinejad M (2021). The impact of Covid-19 outbreak on nosocomial infection rate: a case of Iran. Can J Infect Dis Med Microbiol.

[10] Su C, Zhang Z, Zhao X, Peng H, Hong Y, Huang L (2021). Changes in prevalence of nosocomial infection pre-and post- Covid-19 pandemic from a Tertiary Hospital in China. BMC Infect Dis.

[11] Wee LEI, Conceicao EP, Tan JY, Magesparan KD, Amin IBM, Ismail BBS (2021). Unintended consequences of infection prevention and control measures during Covid-19 pandemic. Am J Infect Control.

[12] Ghashghaee A, Behzadifar M, Azari S, Farhadi Z, Bragazzi NL, Behzadifar M (2018). Prevalence of nosocomial infections in Iran: a systematic review and meta-analysis. Med J Islam Repub Iran.

[13] Rosenthal VD, Desse J, Maurizi DM, Chaparro GJ, Orellano PW, Chediack V (2018). Impact of the International Nosocomial Infection Control Consortium's multidimensional approach on rates of ventilator-associated pneumonia in 14 intensive care units in 11 hospitals of 5 cities within Argentina. Am J Infect Control.

[14] Patel PK, Gupta A, Vaughn VM, Mann JD, Ameling JM, Meddings J (2018). Review of strategies to reduce central line-associated bloodstream infection (CLABSI) and catheter-associated urinary tract infection (CAUTI) in adult ICUs. J Hosp Med.

[15] Magiorakos A-P, Srinivasan A, Carey RB, Carmeli Y, Falagas M, Giske C (2012). Multidrug-resistant, extensively drug-resistant and pandrug-resistant bacteria: an international expert proposal for interim standard definitions for acquired resistance. Clin Microbiol Infect.

[16] Baccolini V, Migliara G, Isonne C, Dorelli B, Barone L, Giannini D (2021). The impact of the Covid-19 pandemic on healthcare-associated infections in intensive care unit patients: a retrospective cohort study. Antimicrob Resist Infect Control.

[17] McMullen KM, Smith BA, Rebmann T (2020). Impact of SARS-CoV-2 on hospital acquired infection rates in the United States: predictions and early results. Am J Infect Control.

[18] LeRose J, Sandhu A, Polistico J, Ellsworth J, Cranis M, Jabbo L (2020). The impact of coronavirus disease 2019, (Covid-19) response on central-line–associated bloodstream infections and blood culture contamination rates at a tertiary-care center in the Greater Detroit area. Infect Control Hosp Epidemiol.

[19] Maes M, Higginson E, Pereira-Dias J, Curran MD, Parmar S, Khokhar F (2021). Ventilator-associated pneumonia in critically ill patients with Covid-19. Crit Care.

[20] Al-Hasan MN, Winders HR, Bookstaver PB, Justo JAJA (2019). Direct measurement of performance: a new era in antimicrobial stewardship. Antibiotics.

[21] Leblebicioglu H, Erben N, Rosenthal VD, Atasay B, Erbay A, Unal S (2014). International Nosocomial Infection Control Consortium (INICC) national report on device-associated infection rates in 19 cities of Turkey, data summary for 2003–2012. Ann Clin Microbiol Antimicrob.

