# Supplementary material for: Unintended impact of COVID-19 pandemic on the rate of catheter related nosocomial infections and incidence of multiple drug resistance pathogens in three intensive care units not allocated to COVID-19 patients in a large teaching hospital
Source: BMC Infect Dis. 2023 Jan 6;23:11. doi: 10.1186/s12879-022-07962-7 (PMC9821351; doi:10.1186/s12879-022-07962-7)
Supplement: Supplementary file 1 — Additional file 1. Additional figures. [file 12879_2022_7962_MOESM1_ESM.docx]

**Additional file 1. Additional figures**

Pre-pandemic, the rate of Klebsiella VAP was 2.38 cases per 1,000 device-days (9 cases; 3,787 device-days) and, in pandemic period, was 1.43 cases per 1,000 device-days (3 cases; 2,096 device-days) that the difference between them were not statistically significant (IRR = 1.66, 95% CI = 0.41-9.53, P =0.442).

Pre-pandemic, the rate of Pseudomonas-aeruginosa VAP was 1.06 cases per 1,000 device-days (4 cases; 3,787 device-days) and, in pandemic period, was 0.48 cases per 1,000 device-days (1 cases; 2,096 device-days) that the difference between them were not statistically significant (IRR = 2.21, 95% CI = 0.22-109.03, P =0.466).

Pre-pandemic, the rate of Escherichia-coli VAP was 0.53 cases per 1,000 device-days (2 cases; 3,787 device-days) and, in pandemic period, was 0.48 cases per 1,000 device-days (1 cases; 2,096 device-days) that the difference between them were not statistically significant (IRR = 1.11, 95% CI = 0.06-65.31, P =0.934).
